# Modelling lung and muscle oxygen diffusion capacities from sea-level to Mount Everest

**DOI:** 10.1038/s41598-025-32441-9

**Published:** 2026-03-02

**Authors:** Nicolas Bourdillon, Giorgio Manferdelli, Antoine Raberin, Grégoire P. Millet

**Affiliations:** ISSUL, Institute of Sport Sciences, University of Lausanne, bâtiment Synathlon, Lausanne, 1015 Switzerland

**Keywords:** Exercise, O_2_ transport, Modelling, Diffusion, Lung, Muscle, Altitude, Climate sciences, Physiology

## Abstract

Lung and muscle oxygen diffusion capacities (DLO_2_ and DMO_2_, respectively) are difficult to measure at maximal-intensity exercise and at altitude and they are scarcely reported in the literature, yet they are key components of the O_2_ transport cascade. The goal of the present study was to compute DLO_2_ and DMO_2_ at simulated increasing altitudes between sea-level and Mount Everest. Literature data were compiled to compute DLO_2_ and DMO_2_ at maximal exercise using a forward iterative algorithm. These computations were repeated every 250 m of increasing altitude between seal level and the altitude of Mount Everest. Computed DLO_2_ increased from sea-level to 5500 m and then decreased to the altitude of Mount Everest; yet remaining higher than sea-level values. DMO_2_ increased from sea-level to 3500 m and then progressively decreased to values lower than sea-level. The computed variations in DLO_2_ and DMO_2_ fit with the ability of the lung and muscle to increase their diffusion capacity at altitude, which seemingly indicates an existing diffusion capacity reserve. The muscle reserve seems depleted at a lower altitude than the lung reserve. The clinical relevance of the proposed model requires further investigation.

## Introduction

Since Sir Archibald V. Hill raised the question of the determinants of peak oxygen consumption ($\:\dot{\mathrm{V}}$O_2_peak) in 1923^1^, there has been a heated debate on the underlying mechanisms^2, 3^. For long, it was considered that the changes in $\:\dot{\mathrm{V}}$O_2_peak (due to training, ageing, altitude exposure, etc.) were driven exclusively by convective factors and the O_2_ supply ($\:\dot{\mathrm{Q}}$aO_2_); *de facto* considering that the O_2_ diffusion capacity of the muscle (DMO_2_, *a.k.a diffusing capacity*) was a negligible limiting factor. However, thirty years ago, Peter D. Wagner modelled the oxygen transport through the human body using data from Operation Everest II and demonstrated the presence of a finite and limited DMO_2_, thus explaining the presence of small amount of O_2_ in the venous effluent blood at $\:\dot{\mathrm{V}}$O_2_peak^4^.

Since Operation Everest II, it has been demonstrated that a substantial part of the increase in $\:\dot{\mathrm{V}}$O_2_peak is attributable to an increase in DMO_2_ rather than $\:\dot{\mathrm{Q}}$aO_2_ in response to exercise training^5^. This finding has clinical applications; e.g. in heart failure patients, for whom a reduced DMO_2_ significantly limits $\:\dot{\mathrm{V}}$O_2_peak^6, 7^.

Reported DMO_2_ during maximal-intensity exercise was shown to either increase^8^ or decrease^9, 10^ in altitude. These discrepancies may be explained primarily by the various altitude levels and the various methods for measuring cardiac output, leg blood flow, oxygen consumption, blood haemoglobin concentration and other variables required for computation. To date, previous studies^8, 9, 10, 11^ reported DMO_2_ only at one or two altitude levels (typically 5,000 m or above) and, to our knowledge, there is no report of DMO_2_ variations across a large range of increasing altitude between sea-level and Mount Everest.

A classical assumption in altitude physiology is that the O_2_ partial pressure at the mitochondria level (PmitO_2_) is around 2–3 mmHg at maximal-intensity exercise and is considered negligible compared with the PO_2_ at the upstream steps of the O_2_ cascade. Therefore, PmitO_2_ is set at zero in the computations^4^. However, the actual pressure at the mitochondria in a working muscle may be around 1–5 mmHg as measured in situ in dog’s *gracilis* muscle^12^. Values of PmitO_2_ between 0 and 20 mmHg have been proposed in previous models, with the conclusion that it likely played a minor role in the total O_2_ flux resistance^9^. Another model^13, 14^ attributed to the peripheral factors (including PmitO_2_) a potential greater role - although remaining of minor importance - in the O_2_ flux resistance compared the model presented hereafter.

Along with DMO_2_, the pulmonary O_2_ diffusion capacity (DLO_2_) is also an essential part of O_2_ transport modelling through the human body. At altitude, DLO_2_ is believed to contribute to limit oxygen diffusion in the lung^15, 16^ but similarly to DMO_2_, studies consistently reporting DLO_2_ between sea-level and the altitude of Mount Everest are lacking.

Measuring these diffusion components (DLO_2_ and DMO_2_) is technically more difficult than measuring the convective components, especially in field studies and at maximal exercise. Modelling these diffusion capacity changes during maximal exercise is therefore valuable, particularly at altitude, where the studies reporting DLO_2_ and DMO_2_ are scarce and based on small databases. The goal of the present study was therefore to compute DLO_2_ and DMO_2_ at simulated increasing altitudes between sea-level and Mount Everest (8,848 m), based on literature and laboratory data.

## Methods

### Modelling the O_2_ transport

The present model is based on the classical mass conservation equations for O_2_. The equations were written as they were used in the computer code to achieve the numerical solution with i representing time and/or distance (i.e. the iterative computation along the blood capillary). The iterative computations used herein compute blood O_2_ content as blood flows through the capillary, i can therefore be considered as time (e.g. blood content 1 ms, 2 ms 3 ms etc. into the capillary) or as distance (e.g. blood content at 1 nm, 2 nm, 3 nm into the capillary). The left-hand part of the present Eqs. 1 and 3 represents convective transport of O_2_ by the blood according to Fick’s equation, whereas the right-hand part represents O_2_ diffusion from the capillary to the surrounding tissues according to Fick’s principle. Equation 1 represents O_2_ mass conservation equation for the muscular circulation and was solved for DMO_2_ (Eq. 1)^17, 18^.

1 $$\:\dot{\mathrm{Q}}\:.\left(\mathrm{C}\mathrm{c}\mathrm{a}\mathrm{p}\mathrm{m}{\mathrm{O}}_{2\:\left(\mathrm{i}+1\right)}-\:\mathrm{C}\mathrm{c}\mathrm{a}\mathrm{p}\mathrm{m}{\mathrm{O}}_{2\:\left(\mathrm{i}\right)}\right)=\:\mathrm{D}\mathrm{M}{\mathrm{O}}_{2}\:.(\mathrm{P}\mathrm{c}\mathrm{a}\mathrm{p}\mathrm{m}{\mathrm{O}}_{2\:\left(\mathrm{i}\right)}-\mathrm{P}\mathrm{m}\mathrm{i}\mathrm{t}{\mathrm{O}}_{2})$$

where $\:\dot{\mathrm{Q}}$ is cardiac output in l_blood_/min; CcapmO_2_ is capillary blood O_2_ content, which decreases with time when blood flows through the muscle capillary (lO_2_/l_blood_); $\:\mathrm{D}\mathrm{M}{\mathrm{O}}_{2}$ is the muscle O_2_ diffusion capacity in lO_2_/min/mmHg; $\:\mathrm{P}\mathrm{c}\mathrm{a}\mathrm{p}\mathrm{m}{\mathrm{O}}_{2}$ is PO_2_ in the capillary, which decreases when blood flows through (mmHg); and $\:\mathrm{P}\mathrm{m}\mathrm{i}\mathrm{t}{\mathrm{O}}_{2}$ is PO_2_ at the mitochondria in mmHg. CcapmO_2_ is classically representing blood O_2_ content in the capillary, which comprises O_2_ bound to haemoglobin and O_2_ dissolved in plasma, and can be written as^19^:

2 $$\:\mathrm{C}\mathrm{c}\mathrm{a}\mathrm{p}\mathrm{m}{\mathrm{O}}_{2}=1.34\:\cdot\:\left[\mathrm{H}\mathrm{b}\right]\cdot\:\mathrm{S}\mathrm{c}\mathrm{a}\mathrm{p}{\mathrm{O}}_{2}/100+\mathrm{P}\mathrm{c}\mathrm{a}\mathrm{p}{\mathrm{m}\mathrm{O}}_{2}\:\cdot\:0.31$$

Where 1.34 is haemoglobin carrying capacity for O_2_ in mlO_2_/g_Hb_, [Hb] is blood haemoglobin concentration (g_Hb_/l_blood_) and ScapO_2_ is blood oxygen saturation (%) in the capillary. Equation 1 is usually solved with iterative computation techniques^11^, typically using Fibonacci’s method^18^.

The O_2_ mass conservation equation for the pulmonary circulation is as follows:

3 $$\:\dot{\mathrm{Q}}\:.\left(\mathrm{C}\mathrm{c}\mathrm{a}\mathrm{p}\mathrm{l}{\mathrm{O}}_{2\:\left(\mathrm{i}+1\right)}-\:\mathrm{C}\mathrm{c}\mathrm{a}\mathrm{p}\mathrm{l}{\mathrm{O}}_{2\:\left(\mathrm{i}\right)}\right)=\:\mathrm{D}\mathrm{L}{\mathrm{O}}_{2}\:\:.(\mathrm{P}\mathrm{A}{\mathrm{O}}_{2}-\:\mathrm{P}\mathrm{c}\mathrm{a}\mathrm{p}\mathrm{l}{\mathrm{O}}_{2\:\left(\mathrm{i}\right)})$$

where $\:\dot{\mathrm{Q}}$ is cardiac output in l_blood_/min; CcaplO_2_ is lung capillary O_2_ content, which increases with time when blood flows through the muscle capillary (lO_2_/l_blood_) ; $\:\mathrm{D}\mathrm{L}{\mathrm{O}}_{2}$ is the pulmonary O_2_ diffusion capacity (lO_2_/min/mmHg); $\:\mathrm{P}\mathrm{A}{\mathrm{O}}_{2}$ is PO_2_ in the lung alveoli (mmHg); and $\:\mathrm{P}\mathrm{c}\mathrm{a}\mathrm{p}\mathrm{l}{\mathrm{O}}_{2}$ is PO_2_ in the lung capillary, which increases when blood flows through (mmHg). CcaplO_2_ is classically representing blood O_2_ content in the lung capillary, which comprises O_2_ bound to haemoglobin and O_2_ dissolved in plasma (Eq. 2). Similarly to Eq. 1, Eq. 3 is usually solved using Fibonacci’s method^18^.

### Data compilation

Data from the literature were compiled for $\:\dot{\mathrm{V}}{\mathrm{O}}_{2}$, $\:\dot{\mathrm{Q}}$, PAO_2_, PaO_2_, SaO_2_, Hb and $\:\mathrm{P}\stackrel{-}{\mathrm{v}}{\mathrm{O}}_{2}$ at maximal exercise at various altitude ranging from sea-level to the altitude of Mount Everest using the articles and values listed in Table 1 (appended at the end of the present article). The compiled data were fitted using the Leave-One-Out cross-validation method^20^ for determining the best polynomial order for each parameter. Subsequently, the fits were interpolated for each 250-m step of altitude.

**Table 1:** Compilation of maximal exercise data from the literature, rangin from sea level data to Mount Everest. This data was used as input of the mathematical model..

Name - date	References	Altitude (m)	Patm (mmHg)	PiO_2_ (mmHg)	$\:\dot{\mathrm{V}}{\mathrm{O}}_{2}\mathrm{p}\:\:$(ml/min)	$\:\dot{\mathrm{Q}}$(l/min)	PAO_2_ (mmHg)	PaO_2_ (mmHg)	SaO_2_ (mmHg)	[Hb] (g/l)	$\:\mathrm{P}{\stackrel{-}{\mathrm{v}}}{\mathrm{O}}_{2}$(mmHg)	*n* (subjects)
Somervell 1925	^58^	8848	250	42.5	–	–	–	–	–	20.6	–	5
Luft 1941	^57^	8125	267	48	–	–	–	–	–	20	–	5
Pugh 1954	^59^	8153	267	46	–	–	–	–	–	20.3	–	8
Pugh 1954	^60^	8848	240	43	–	–	–	–	–	20.9	–	12
Matthews 1955	^61^	8580	244	44	–	–	–	–	–	20.3	–	6
Pace 1956	^62^	8458	244	44	–	–	–	–	–	–	–	10
Brendel 1956	^63^	8848	240	43	–	–	–	–	–	21.1	–	6
Pugh 1962	^64^	5800	364	66.4	–	–	–	–	–	19.6	–	8
West 1962	^65^	5800	380	80	2260	–	55	35	67	–	–	1
		5800	380	80	1990	–	52	30	58	–	–	1
		5800	380	80	2090	–	53	–	45	–	–	1
		5800	380	80	2140	–	52	–	45	–	–	1
		5800	380	80	2190	–	55	–	45	–	–	1
Faulkner 1966	^66^	0	760	160	3838	–	–	–	–	14.5	–	4
		2300	560	117.6	3576	–	–	–	–	16	–	
Cerretelli 1976	^67^	0	760	150	3213	–	–	–	97.8	15	–	35
		5350	384	70.6	2260	–	–	–	77.4	20.6	–	
West 1983	^68^	0	750	147.1	4630	–	119	–	95	–	–	15
		6300	351	63.7	2310	–	52	31	59	–	–	
		8050	280	48.5	1450	–	39	27	55	–	–	
		8848	250	42.5	1070	–	37	27	46	–	–	
OEII 1985	^53^	0	760	160	3710	24.2	115	87	95.5	–	18.5	6
		6500	300	63	2098	20.3	46	34	61.9	–	12.4	
		7622	233	49	1450	15.9	40.8	33.1	58.3	–	14.1	
		8100	205	43	1177	16.1	34.8	27.6	50.5	–	13.8	
OEII 1985 2	^35^	0	760	150	3980	27.2	–	94	93	–	–	8
		6100	347	63	2060	20	–	32	47	–	–	
		7620	282	53	1770	17.7	–	32	39	–	–	
		8848	240	43	1170	14.3	–	27	35	–	–	
Comex 1997	^ 69^	0	760	160	4210	–	–	–	97	–	–	8
		6000	370	78	1684	–	–	–	72	17.9	–	
Calbet 2002	^70^	5260	361	76	–	–	–	46	73.3	19.1	–	8
Richalet 2004	^39^	0	760	150	–	15.6	112.3	89.1	–	–	–	12
		4350	439	82	–	14.6	69.9	39.5	–	–	–	
Mollard 2007 1	^71^	0	760	150	3027	–	124.1	99.5	–	–	24.9	8
		1000	674	134	2936	–	103.1	79.3	–	14	23.4	
		2500	561	112	2754	–	84.2	58.8	–	–	20.6	
		4500	430	86	2495	–	61.6	39.3	–	–	14.9	
Mollard 2007 2	^72^	0	760	150	4683	–	120.1	82.6	–	–	–	8
		1000	674	134	4483	–	105.4	66.5	–	14	–	
		2500	561	112	4126	–	84.7	49.4	–	–	–	
		4500	430	86	3318	–	58.4	34.1	–	–	–	
Mollard 2007 3	^73^	0	760	150	2009	–	122.1	98	–	14.2	26.3	8
		1000	674	134	1956	–	105.1	81.6	–	14	21.8	
		2500	561	112	1860	–	83	59	–	–	18.9	
		4500	430	86	1642	–	57.9	39.2	–	–	15.2	
Mollard 2007 4	^73^	0	760	150	3185	–	118	91.8	–	13.5	–	8
		1000	674	134	3012	–	101.3	72.4	–	14	–	
		2500	561	112	2808	–	80.6	51.3	–	–	–	
		4500	430	86	2345	–	59.3	36.9	–	–	–	
vanHall 2009	^74^	0	760	150	4600	12.3	–	98.8	98.4	13.9	–	6
		4100	455	85.5	3700	8.7	–	58.4	89.7	15.9	–	
Bourdillon 2009 1	^18^	0	760	150	4716	27	–	–	95	–	–	7
		1000	674	134	4514	26.8	–	–	91	14	–	
		2500	561	112	4154	26.9	–	–	82	–	–	
		4500	430	86	3341	25.4	–	–	66	–	–	
Bourdillon 2009 2	^18^	0	760	150	3031	22.2	–	–	96	–	–	8
		1000	674	134	2940	21.6	–	–	94	14	–	
		2500	561	112	2758	21.4	–	–	89	–	–	
		4500	430	86	2499	21.2	–	–	73	–	–	
Bourdillon 2009 3	^75^	0	760	150	3411	23.4	–	–	96	–	–	6
		1000	674	134	3352	23.6	–	–	93	14	–	
		2500	561	112	3241	23.1	–	–	89	–	–	
		4500	430	86	3086	22.6	–	–	77	–	–	
Bourdillon 2009 4	^75^	0	760	150	4351	28	–	–	96	–	–	5
		1000	674	134	3884	27	–	–	93	14	–	
		2500	561	112	3650	27.7	–	–	84	–	–	
		4500	430	86	3183	25.7	–	–	71	–	–	
Mollard 2009	^76^	0	760	150	–	–	–	93.7	94.4	–	–	10
		4500	430	86	–	–	–	40.9	69.2	–	–	
Faiss 2014 1	^77^	0	760	150	3969	–	–	–	91.1	–	–	5
		3000	526	110	3213	–	–	–	76.4	–	–	
Faiss 2014 2	^77^	0	760	150	3169	–	–	–	94.3	–	–	5
		3000	526	110	2801	–	–	–	82.3	–	–	
Raberin 2021	^78^	0	760	150	4816	29.3	–	–	95	15.4	–	17
		2400	560	117.6	4117	27.7	–	–	83.6	16.3	–	
Manferdelli 2023	^79^	0	760	150	3930	13.3	–	–	96	–	–	34
		3375	503	106	2840	11.6	–	–	83	–	–	

Some studies measured data from both venous and arterial blood whereas other studies computed venous O_2_ content as described below, following Fick’s method.

#### Computations for venous content

The following computations were performed at maximal exercise, based on data extracted from the studies and values listed in Table 1 when the values were not directly reported in the concerned articles.

- $\:\mathrm{C}\mathrm{a}{\mathrm{O}}_{2}=1.34\:\cdot\:\left[\mathrm{H}\mathrm{b}\right]\cdot\:\frac{\mathrm{S}\mathrm{a}{\mathrm{O}}_{2}}{100}+\mathrm{P}\mathrm{a}{\mathrm{O}}_{2}\:\cdot\:0.31$ Eq. 4, given measured values of [Hb], SaO_2_ and PaO_2_ from arterial or arterialized blood samples. Value of plasma oxygen solubility taken according to Christmas et al.^21^.
- $\:\mathrm{C}\stackrel{-}{\mathrm{v}}{\mathrm{O}}_{2}=\mathrm{C}\mathrm{a}{\mathrm{O}}_{2}-\:\frac{\dot{\mathrm{V}}{\mathrm{O}}_{2}}{\dot{\mathrm{Q}}}$ Eq. 5, given measured values of $\:\dot{\mathrm{V}}{\mathrm{O}}_{2}$ and $\:\dot{\mathrm{Q}}$. $\:\mathrm{C}\stackrel{-}{\mathrm{v}}{\mathrm{O}}_{2}$ is mixed venous oxygen content.
- $\:\mathrm{S}\stackrel{-}{\mathrm{v}}{\mathrm{O}}_{2}=\frac{\mathrm{C}\stackrel{-}{\mathrm{v}}{\mathrm{O}}_{2}}{\left[\mathrm{H}\mathrm{b}\right]\cdot \:1.34}$ Eq. 6, $\:\mathrm{S}\stackrel{-}{\mathrm{v}}{\mathrm{O}}_{2}$ is the mixed venous oxygen saturation.
- $$\:\mathrm{P}\stackrel{-}{\mathrm{v}}{\mathrm{O}}_{2}=\mathrm{p}50\:\cdot\:\:{\left(\frac{\mathrm{S}\stackrel{-}{\mathrm{v}}{\mathrm{O}}_{2}}{1-\mathrm{S}\stackrel{-}{\mathrm{v}}{\mathrm{O}}_{2}}\right)}^{\raisebox{1ex}{$1$}\!\left/\:\!\raisebox{-1ex}{$\mathrm{N}$}\right.}$$ Eq. 7, with standard p50 of 26.6 mmHg^22^ and the N of Hill of 2.7^23, 24^. P$$\:\stackrel{-}{\mathrm{v}}{\mathrm{O}}_{2}$$ is the mixed venous oxygen pressure.

The second step of modelling was achieved by using the values of $\:\dot{\mathrm{V}}{\mathrm{O}}_{2}$, $\:\dot{\mathrm{Q}}$, $\:\mathrm{C}\mathrm{a}{\mathrm{O}}_{2}$, PaO_2_, $\:\mathrm{C}\stackrel{-}{\mathrm{v}}{\mathrm{O}}_{2}$ and $\:\mathrm{P}\stackrel{-}{\mathrm{v}}{\mathrm{O}}_{2}$, and applying Fibonacci’s method. The outcomes were DMO_2_ and DLO_2_ in mlO_2_/min/mmHg computed at maximal exercise. Computations for DMO_2_ were repeated for PmitO_2_ values ranging from 0 to each individual’s $\:\mathrm{P}\stackrel{-}{\mathrm{v}}{\mathrm{O}}_{2}$ (see below). Both DMO_2_ and DLO_2_ were computed for every 250-m of increasing altitude from sea-level to Mount Everest.

### Solving the equation: fibonacci’s method

**Fig. 1:**
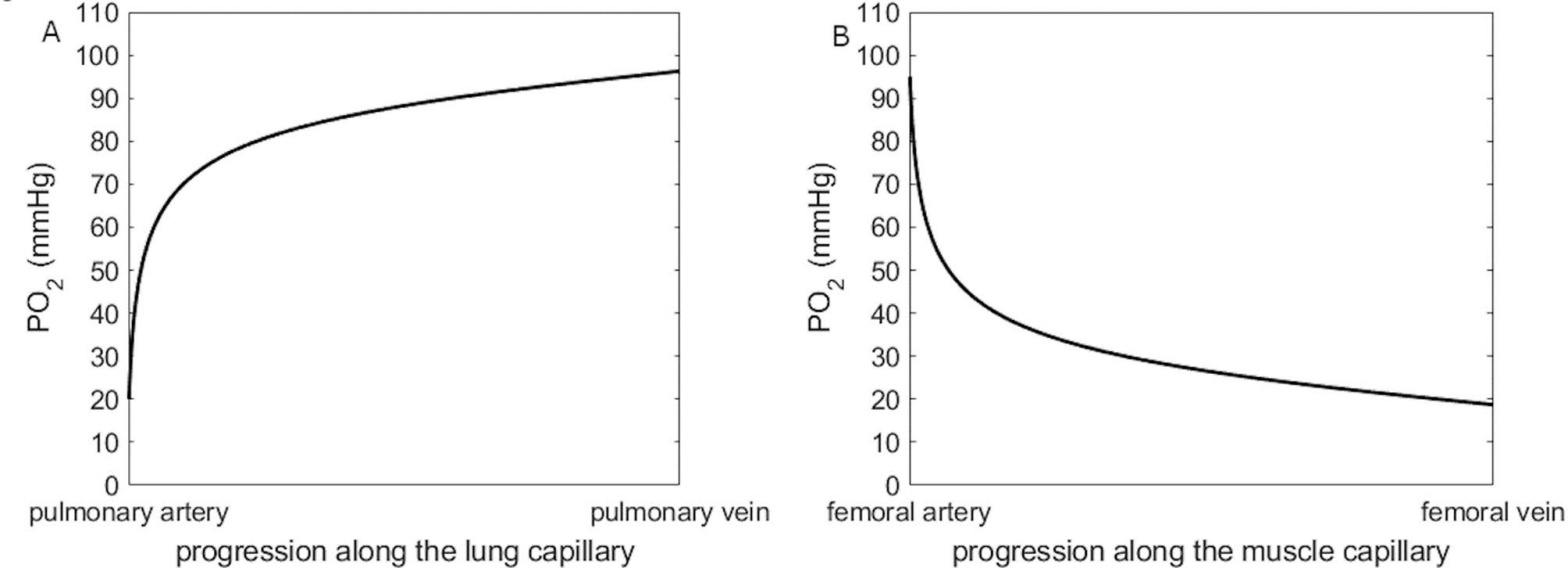
Partial oxygen pressure (PO_2_) in the blood varying along its progression through the lung (panel **A**) and muscle (panel **B**) capillary when solving the mass conservation equations (Eq. 3 and Eq. 1 respectively).

Blood O_2_ content at the entry and the exit of a capillary is known. In the muscle, PaO_2_ and CaO_2_ are the entry data (arterial end of the capillary) whereas $\:\mathrm{P}\stackrel{-}{\mathrm{v}}{\mathrm{O}}_{2}$ and $\:\mathrm{C}\stackrel{-}{\mathrm{v}}{\mathrm{O}}_{2}$ are the exit data (venous end of the capillary). Given the blood flow and PmitO_2_, PcapO_2_ can be computed. Fibonacci’s method lies in computing PO_2_ all along the muscle capillary, presently divided in 1000 successive compartments, using a random DMO_2_. Then compute the difference between PO_2_ at the venous end of the capillary and the measured P$\:\stackrel{-}{\mathrm{v}}$O_2_. Renew the computation with another DMO_2_, if the difference between PO_2_ at the venous end and measured P$\:\stackrel{-}{\mathrm{v}}$O_2_ is smaller than previously, then the latter DMO_2_ is preferred. If these computations are repeated several times (e.g., 1000 times in the present study), the difference between PO_2_ at the venous end and measured P$\:\stackrel{-}{\mathrm{v}}$O_2_ becomes less than 0.1% and the associated computed DMO_2_ is estimated as a reliable surrogate of the true DMO_2_, with a unique solution^18^. Figure 1, panel A shows an example of PO_2_ varying along the lung capillary when Eq. 3 is solved for DLO_2_; and Fig. 1B shows varying PO_2_ along a muscle capillary when Eq. 1 is solved for DMO_2_.

Fibonacci’s method can be repeated for a range of PmitO_2_, which can logically be selected from zero (i.e., the generally accepted assumption by physiologists) to P$\:\stackrel{-}{\mathrm{v}}$O_2_. PmitO_2_ cannot be greater than P$\:\stackrel{-}{\mathrm{v}}$O_2_ to ensure an O_2_ pressure gradient from the capillary to the mitochondria compatible with O_2_ diffusion. Such method implies that PmitO_2_ represents an “average” PmitO_2_ gathering various tissues; e.g., in active muscles where O_2_ demand is maximal and thus PmitO_2_ is likely as low as 0–1 mmHg as well as in skin where O_2_ demand is low and thus PmitO_2_ as high as 60 mmHg^25^.

The computation of DLO_2_ follows the same steps than the computation of DMO_2_ except that the entry point is determined by $\:\mathrm{P}\stackrel{-}{\mathrm{v}}{\mathrm{O}}_{2}$ and $\:\mathrm{C}\stackrel{-}{\mathrm{v}}{\mathrm{O}}_{2}$ (pulmonary artery end of the capillary) and the exit point is determined by PaO_2_ and CaO_2_ (pulmonary vein end of the capillary). The pressure driving the gradient is PAO_2_ (instead of PmitO_2_ in the muscle).

All computations were performed with a standard p50 of 26.6 mmHg except otherwise specified. Computations were repeated with various p50 to test the sensitivity of the computed DMO_2_ to this parameter. Unfortunately, p50 is scarcely reported at maximal-intensity exercise in the literature and therefore could not be reliably fitted using the Leave-One-Out method.

### Sensitivity of fibonacci’s model

The sensitivity of Fibonacci’s model to the other parameters was assessed by applying changes of -20%, -10%, -5%, -1%, + 1%, + 5%, + 10%, and + 20% in $\:\dot{\mathrm{Q}}$, $\:\mathrm{P}\mathrm{A}{\mathrm{O}}_{2}$, PaO_2_, $\:\mathrm{P}\stackrel{-}{\mathrm{v}}{\mathrm{O}}_{2}$, and Hb, respectively. The percent changes in DLO_2_ and DMO_2_ were computed for each individual and at each altitude. This sensitivity computation aimed at determining the potential altitude-induced changes and the influence of each of these input parameters on the two diffusion steps.

### Statistics

The effect of altitude on DLO_2_ and DMO_2_was determined by a one-way ANOVA across each 250-m interval. Multiple comparisons were made with the Tukey post hoc test. A two-way ANOVA with a Tukey post-hoc test was performed to assess potential interaction effects between altitude levels and PmitO_2_ values on the one hand and between altitude levels and p50 values on the other hand. Significance compared to sea-level, the maximum computed value, and the value computed at the altitude of Mount Everest are reported.

## Results

**Fig. 2:**
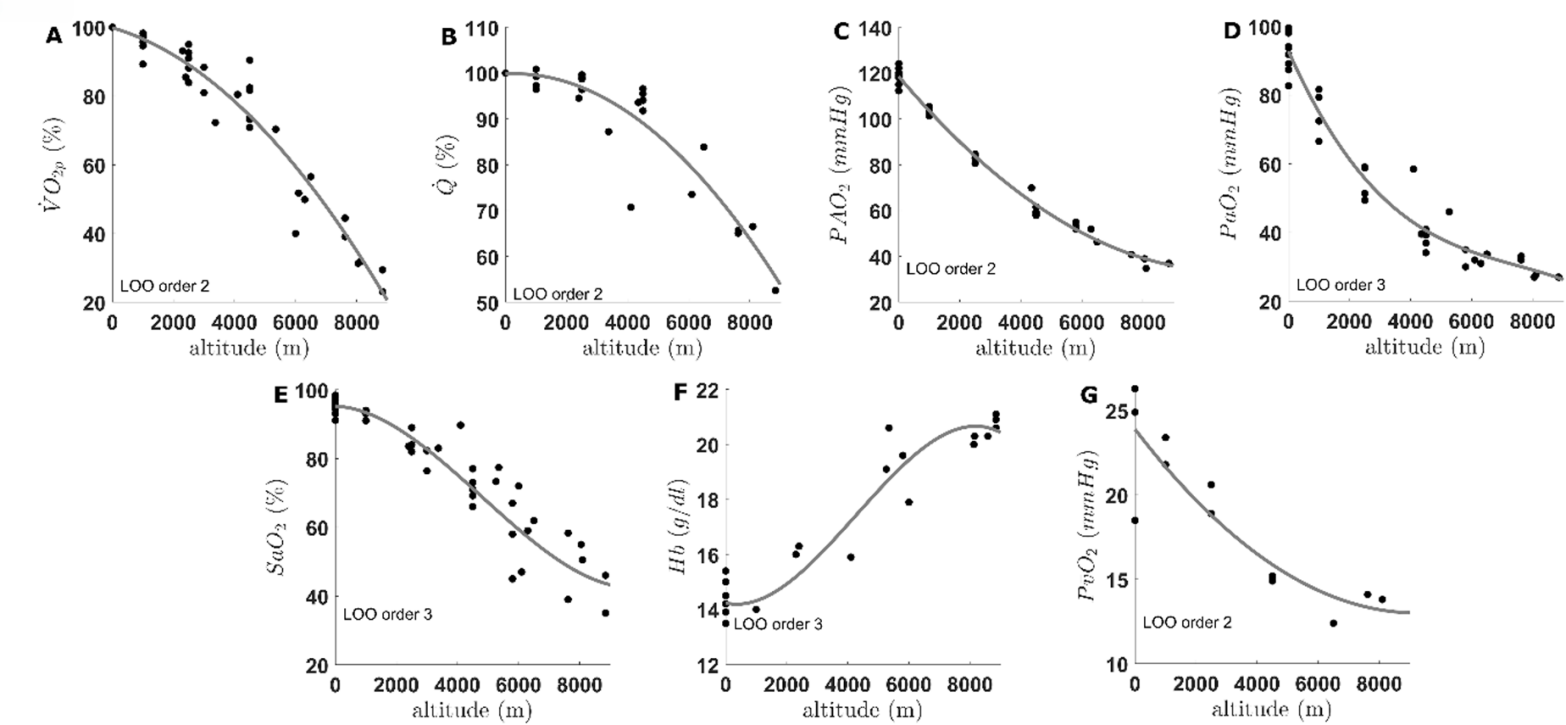
Panel (**A**): Second order polynomial fit for the decrease in peak oxygen consumption ($\:\dot{\mathrm{V}}$O_2_p) expressed in percentage of sea-level; panel (**B**): second order polynomial fit for the decrease in peak cardiac output ($\:\dot{\mathrm{Q}}$) expressed in percentage of sea-level; panel (**C**): second order polynomial fit for the decrease in alveolar oxygen pressure (PAO_2_); panel (**D**): third order polynomial fit for the decrease in arterial oxygen pressure (PaO_2_); panel (**E**): third order polynomial fit for the decrease in arterial oxygen saturation (SaO_2_); panel (**F**): third order polynomial fit for the increase in arterial blood haemoglobin concentration (Hb); panel G: second order polynomial fit for the decrease in mixed venous oxygen pressure (P$\:\stackrel{-}{\mathrm{v}}$O_2_). LOO order is the order given by the Leave-One-Out method and used for the fits. Fits were interpolated for every 250-m step of increasing altitude between sea-level (0 m) and Mount Everest (8848 m). a: *p* < 0.05 for difference with sea-level value, b: *p* < 0.05 for difference with maximal value, c: *p* < 0.05 for difference with Mount Everest value.

The fits of the data from the literature are displayed on Fig. 2 (panel A: $\:\dot{\mathrm{V}}$O_2_peak; panel B: $\:\dot{\mathrm{Q}}$; panel C: PAO_2_; panel D: PaO_2_; panel E: SaO_2_; panel F: Hb; panel G:$\:\:\mathrm{P}\stackrel{-}{\mathrm{v}}{\mathrm{O}}_{2}$) along with the order of the fitted polynomial. The Leave-One-Out cross-validation method resulted in a second order polynomial for all parameters except for PaO_2_, SaO_2_ and Hb which resulted in a third order polynomial. The decrease in $\:\dot{\mathrm{V}}{\mathrm{O}}_{2}$ is expressed in percentage from sea-level value and is comparable with previous literature; e.g., around 85% of sea-level value at 2800 m^26^ and around 20% of sea-level value at Mount Everest^27^. The decrease in $\:\dot{\mathrm{Q}}$ is also expressed in percentage from sea-level value, due to the great inter-individual variability whereas other parameters are expressed in their respective units.

**Fig. 3:**
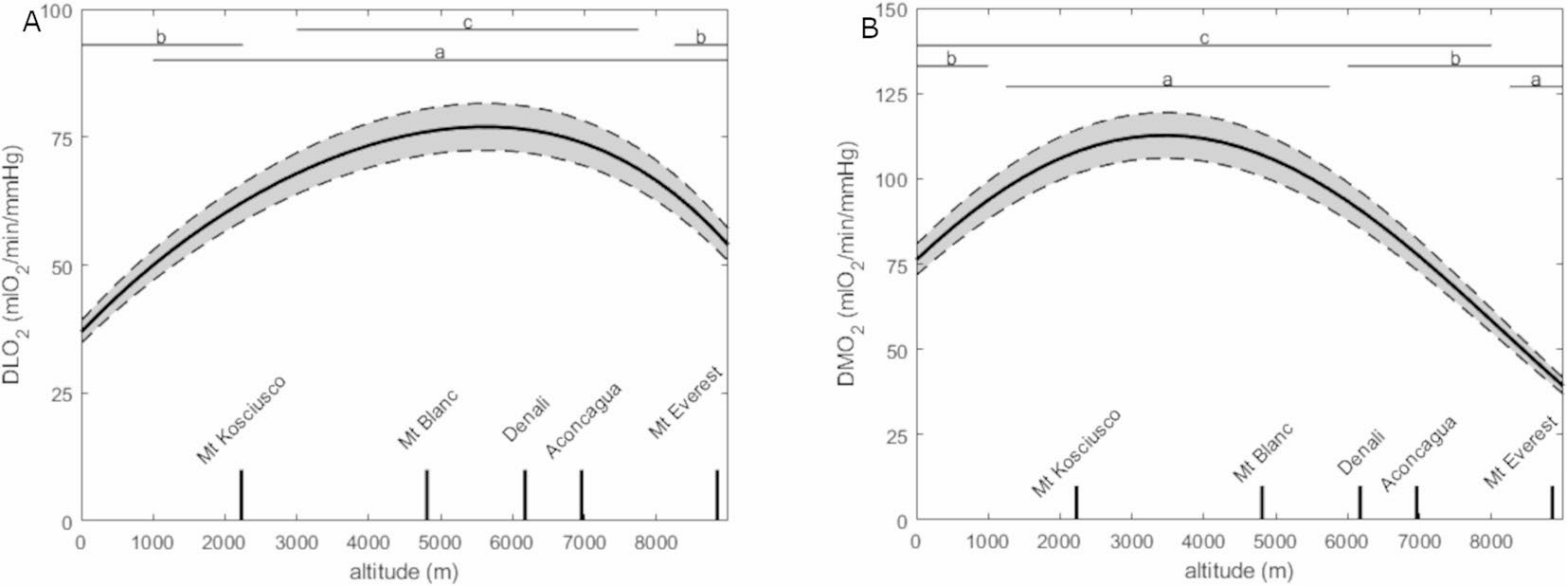
Panel (**A**): Lung oxygen diffusion capacity computed using Fibonacci’s method (DLO_2_) computed each 250 m of altitude between sea-level (0 m) and Mount Everest (8848 m); panel (**B**): muscle oxygen diffusion capacity computed using Fibonacci’s method (DMO_2_) computed for every 250 m of increasing altitude between sea-level (0 m) and Mount Everest (8848 m). Solid line is the average whilst the grey shade between the dashed lines is the 95% confidence interval. a: *p* < 0.05 for difference with sea-level value, b: *p* < 0.05 for difference with maximal value, c: *p* < 0.05 for difference with values computed for Mount Everest.

Both DLO_2_ and DMO_2_ were computed based on sea-level recordings and the polynomials in Fig. 2 to get a value every 250 m of altitude. The results of these computations are displayed in Fig. 3 panel A for DLO_2_ and panel B for DMO_2_, with the 95% confidence interval in grey (computation for PmitO_2_ = 0 mmHg).

DLO_2_ increased progressively up to an altitude of 5500 m and then decreased to the altitude of Mount Everest; yet remaining higher than sea-level values. DLO_2_ was significantly higher than sea-level values from the altitude of 1000 m. It was significantly lower than the maximal value between 0 and 2250 m and above 8250 m. DLO_2_ was significantly higher than Mount Everest value between 3000 and 7750 m.

DMO_2_ increased from sea-level to an altitude of 3500 m and then progressively decreased to a lower value than at sea-level at the altitude of Mount Everest. DMO_2_ was significantly greater than sea-level value between 1250 and 5750 m and significantly lower than sea-level value above 8250 m. It was significantly lower than maximal value between 0 and 1000 m and above 7500 m. DMO_2_ was significantly higher than Mount Everest value between sea-level and 8000 m.

**Fig. 4:**
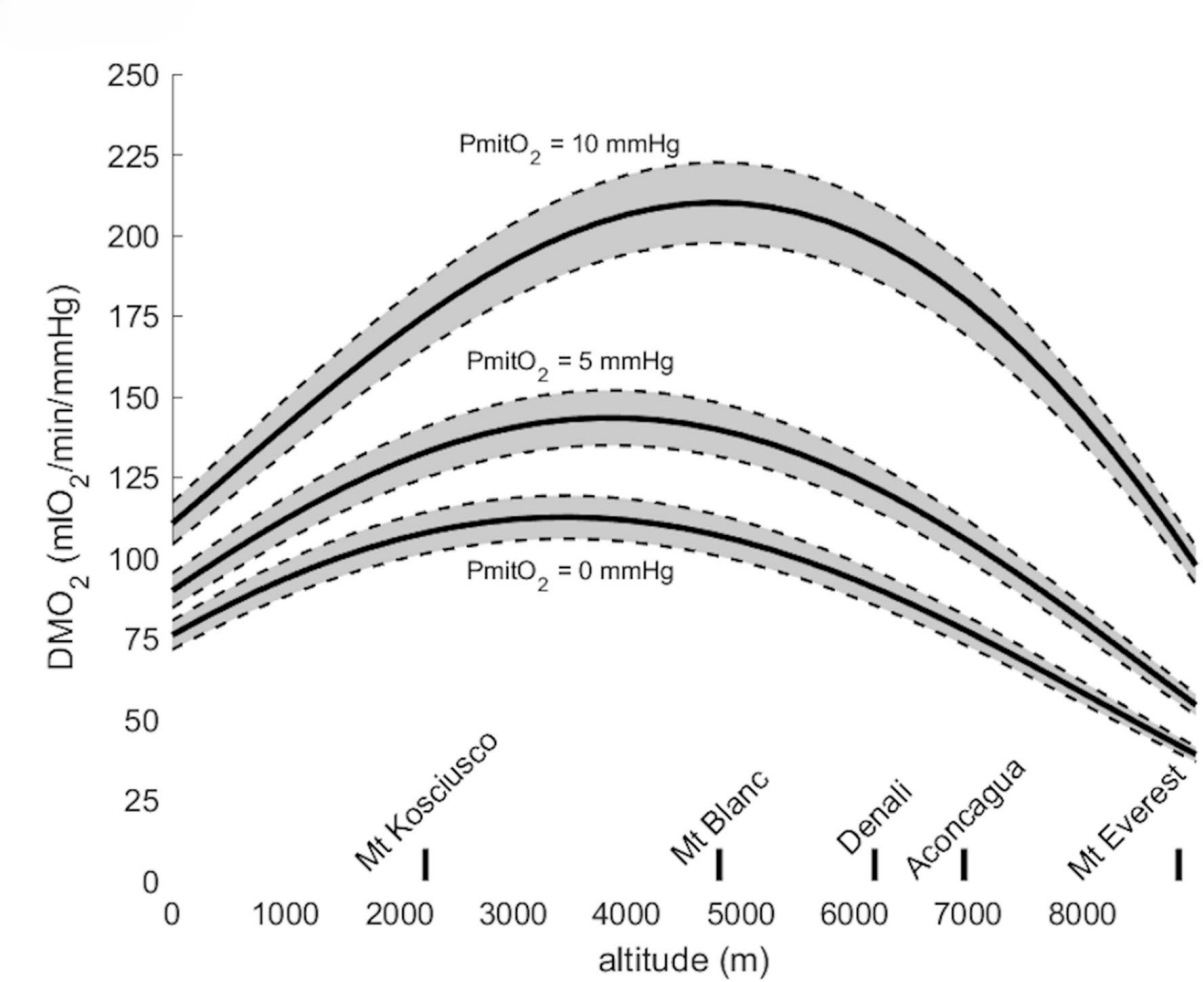
Muscle oxygen diffusion capacity (DMO_2_) computed for mitochondrial PO_2_ (PmitO_2_) of 0, 5 and 10 mmHg across altitude from sea-level to Mount Everest. Shaded area around the solid lines denotes the 95% confidence interval.

Figure 4 shows DMO_2_ computed for PmitO_2_ = 0, 5 and 10 mmHg, respectively. The shape of the curve did not drastically change but the computed values of DMO_2_ were significantly greater with higher PmitO_2_ values. Moreover, the peak values in DMO_2_ occurred at a significantly higher altitude of 3750 and 4750 m for PmitO_2_ = 5 mmHg and 10 mmHg, respectively, when compared with PmitO_2_ = 0 (3500 m).

**Fig. 5:**
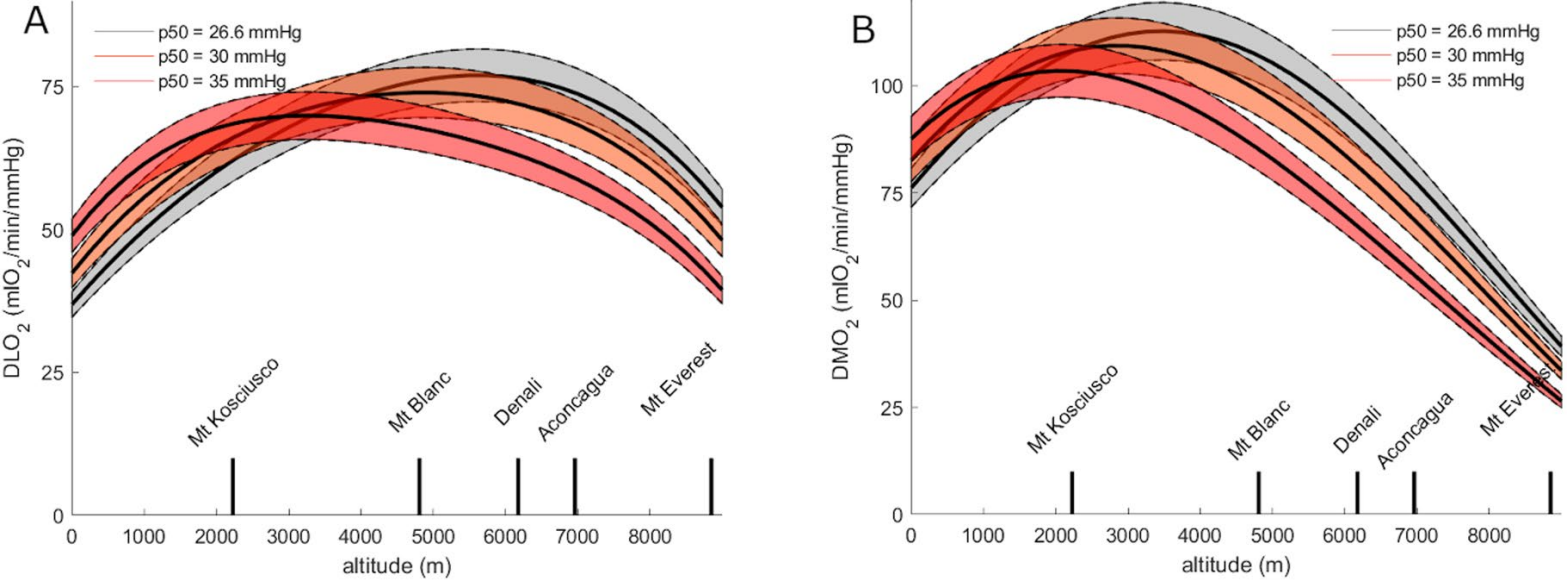
Panel (**A**): Lung oxygen diffusion capacity (DLO_2_); panel (**B**): muscle oxygen diffusion capacity (DMO_2_). Both are computed using three different values of p50 (26.6, 30 and 35 mmHg) across altitude from sea-level to Mount Everest. Shaded area around the solid lines denotes the 95% confidence interval.

Figure 5 shows DLO_2_ and DMO_2_ (panels A and B, respectively) for p50 of 26.6, 30 and 35 mmHg, respectively. The shape of the curves did not drastically change, yet DLO_2_ was higher with p50 of 30 and 35 mmHg, respectively, than with p50 of 26.6 mmHg. DLO_2_ reached a maximal value at 5500 m with p50 of 26.6 mmHg and at a lower altitude of 4750 and 3250 m with p50 of 30 and 35 mmHg, respectively. At the altitude of Mount Everest, DLO_2_ with p50 of 30 and 35 mmHg decreased to lower values than with p50 of 26.6 mmHg. The same observations apply to DMO_2_, with an altitude of 3500 m for its maximal value with p50 of 26.6 instead of 2750 m and 2000 m with p50 of 30 and 35 mmHg, respectively.

**Fig. 6:**
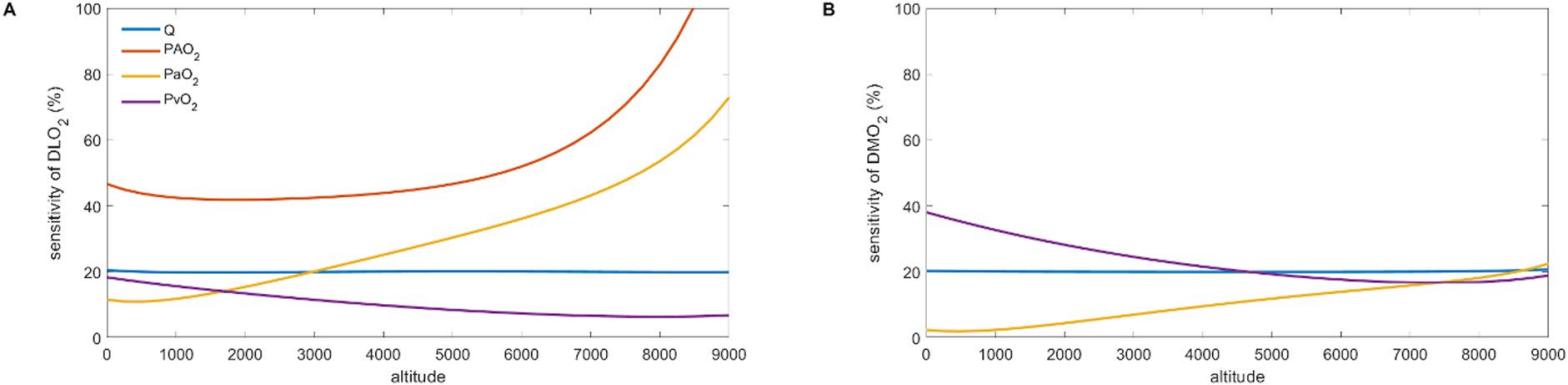
Sensitivity of lung and muscle diffusion capacity (DLO_2_ and DMO_2_, respectively) to cardiac output ($\:\dot{\mathrm{Q}}$), O_2_ partial pressure in the lung alveoli (PAO_2_), arterial O_2_ partial pressure (PaO_2_), mixed venous O_2_ partial pressure (P$\:\stackrel{-}{\mathrm{v}}$O_2_), and blood haemoglobin concentration (Hb) expressed in percentage across altitude from sea-level to Mount Everest.

Figure 6 shows the sensitivity of DLO_2_ and DMO_2_ (panels A and B, respectively) to variations in $\:\dot{\mathrm{Q}}$, $\:\mathrm{P}\mathrm{A}{\mathrm{O}}_{2}$, PaO_2_, $\:\mathrm{P}\stackrel{-}{\mathrm{v}}{\mathrm{O}}_{2}$, and Hb. The weight of $\:\dot{\mathrm{Q}}$ on the two diffusion coefficients was similar (~ 20%) and did not change with increasing altitude. On the contrary, for DLO_2_ the weight of PaO_2_ was smaller than that of $\:\dot{\mathrm{Q}}$ at sea-level but drastically increased with altitude to reach ~ 70% at the altitude of Mount Everest. The weight of PaO_2_ also increased for DMO_2_ but to a lesser extent, reaching the same weight than $\:\dot{\mathrm{Q}}$ at the altitude of Mount Everest. The weight of $\:\mathrm{P}\stackrel{-}{\mathrm{v}}{\mathrm{O}}_{2}$ was always smaller than that of $\:\dot{\mathrm{Q}}$ for DLO_2_ and decreased at high altitude. This decrease was also observed for the weight of $\:\mathrm{P}\stackrel{-}{\mathrm{v}}{\mathrm{O}}_{2}$ on DMO_2_, but globally $\:\mathrm{P}\stackrel{-}{\mathrm{v}}{\mathrm{O}}_{2}$weighted more on DMO_2_ than DLO_2_. As expected, PAO_2_ only influenced DLO_2_ and was paramount from sea-level to the altitude of Mount Everest. Finally, for clarity, the weight (around 20%) of Hb was not plotted but was comparable to $\:\dot{\mathrm{Q}}$.

## Discussion

The aim of this study was to bring a new piece of information on the diffusion components of the O_2_ cascade at altitude. For this purpose: data compiled from the literature from past expeditions to Mount Everest and in the Andes were used (1) to perform polynomial fits and (2) to compute the lung and muscle O_2_ diffusion capacities during a simulated progressive ascent from sea-level to the altitude of Mount Everest.

Our main results were that the pulmonary diffusion capacity increased progressively up to an altitude of 5500 m and then decreased to the altitude of Mount Everest; yet remaining higher than sea-level values, whereas the muscle diffusion capacity increased from sea-level to an altitude of 3500 m and then decreased to the altitude of Mount Everest to a lower value than sea-level value. These altitudes were determined with PmitO_2_ set at 0 mmHg and p50 set at 26.6 mmHg.

Both muscle and lung diffusion are two essential steps of the O_2_ transport chain through the human body but, unlike the convective steps, they are hardly accessible by a direct or an indirect measurement during maximal exercise. Whilst recent research proposed near infrared spectroscopy (NIRS) as a proxy for muscle venous O_2_ saturation^28^, the nitric oxide / carbon monoxide method (DLNO/DLCO) for evaluating the lung diffusion capacity is hardly possible to perform at maximal exercise and in altitude, due to technical limitations^29^.

### Changes in DLO_2_ and DMO_2_ with increasing altitude levels

The computed DLO_2_ values from sea-level to the altitude of Mount Everest showed an increase up to 5500 m and then a progressive decrease to Mount Everest. These findings are consistent with previous reports on the topic where lung diffusion capacity was found increased at 2235, 4350, 4850, 5000 m and 5135 m^29, 30, 31, 32^, which was proposed as indicative of a lung diffusion capacity reserve (i.e. the capacity of the human body to increase DLO_2_ at maximal exercise in hypoxic conditions). According to the present model, one may speculate that this lung reserve is effective up to ~ 5500 m (remarkably, it is the highest altitude of permanent human life on Earth). At high altitude, this reserve has been associated with capillary distension and recruitment, and a consequence of a hypoxic pulmonary vasoconstriction possibly increasing the pulmonary perfusion pressure^33^. Above 5500 m, according to the present computation, this lung reserve decreases, potentially due to either an excessive pulmonary vasoconstriction that impedes pulmonary circulation, or an O_2_ pressure gradient that becomes so low that even an increasing lung capillary recruitment may not be sufficient, or a combination of these two mechanisms. Also, an increase in blood Hb concentration likely contribute to increase DLO_2_ at altitude. The reaction rate between O_2_ and Hb expressed in mlO_2_.min^− 1^.mmHg^− 1^.ml_blood_^−1^ increases at altitude because of the number of O_2_ binding site in blood increases at altitude, which in turn increases DLO_2_^34^. At extreme altitude this effect is less either because Hb reaches a plateau as illustrated in Fig. 2 panel F (because less time is spent at extreme altitude) or hypoxic pulmonary vasoconstriction increases likely reducing capillary blood volume, or a combination of these two mechanisms. Consistent and occasionally remarkable, development of ventilation-perfusion mismatching with both altitude and exercise have been reported^35, 36^. This mismatching and the pattern of distribution that develops were striking and far exceed the abnormalities found acutely when normal subjects are exposed to severe hypoxic conditions, which supports to the hypothesis that most of the intrapulmonary abnormalities observed on exposure to extreme altitude result from pulmonary interstitial fluid accumulation^35^. The increasing importance of lung diffusion at altitude and at maximal exercise is presently illustrated in Fig. 6 panel A with the increasing importance of PAO_2_ and PaO_2_ when altitude increases which confirms previous literature on the topic^13^. In addition, great inter-individual variability has been reported regarding the alveolar-capillary equilibration (corresponding to DLO_2_ in the present study), notably due to a potential interstitial oedema^37^, pulmonary ventilatory response^38^, pulmonary vasomotion^39, 40^, capillary blood volume^41, 42^, capillary blood velocity^29^, and capillary transit time^43^.

DMO_2_ increased up to an altitude of 3500 m and then decreased to a lower value than sea-level value. This may be indicative of a functional reserve (i.e. the capacity of the human body to increase DMO_2_ at maximal exercise in hypoxic conditions) in muscle O_2_ diffusing capacity^8^. This muscle reserve may be smaller than the lung O_2_ diffusing capacity reserve, or at least maximally depleted at a lower altitude. The functional reserve in muscle O_2_ diffusion capacity previously reported at 5000 m ^8^ is in line with the present model. This diffusion capacity reserve is also compatible with the fact that the O_2_ diffusion is only a minor resistance to O_2_ flux^9, 44^, and that the limitations are rather either convective or at the mitochondria level, but not due to the diffusion itself. Additionally, above 5000 m, there is a loss in muscle mass which may account for most of the decrease in DMO_2_. Finally, changes in PmitO_2_ seem to greatly influence DMO_2_ as illustrated in Fig. 4, yet al.l curves keep an inversed U shape. Progressively decreasing PmitO_2_ with increasing altitude at maximal exercise may result in DMO_2_ shifting from a curve to another, probably resulting in a new inversed U-shaped curve, which peak and position remain to be determined. Previous publications reported direct evidence of a functional reserved of muscle O_2_ diffusing capacity during exercise at high but not extreme altitude^8^, compatible with the present findings.

Variations of both DLO_2_ and DMO_2_ are illustrated in Fig. 5 for fixed p50. Yet, with increasing altitude (and resulting adaptations) p50 will progressively change, which means that the variations of both DLO_2_ and DMO_2_ will likely transition from a curve to another, resulting in a new shape of behaviour which remains to be determined^45^. “*All models are wrong*,* but some are useful”* (George E.P. Box, 1976). Since DMO_2_ depends partly on the oxygen delivery at the peripheral level and therefore on previous stages of the O_2_ cascade, it is schematic to display separately their respective kinetics with increasing altitudes.

At the altitude of Mount Everest, the decrease in muscle O_2_ diffusion capacity may be attributed to the low O_2_ supply including decreased arterial oxygen content and blood flow and the low-pressure gradient from the capillary to the mitochondria; all of which creating a low drive for oxygen diffusion. The decrease in P$\:\stackrel{-}{\mathrm{v}}{\mathrm{O}}_{2}$ seems blunted above 8000 m (Fig. 2 panel G), maybe indicating that all available O_2_ has been extracted from the blood and that muscle O_2_ diffusion has reached its limit, given the low drive for O_2_ diffusion.

Although the present study did not intend to revive the debate on the limiting factors of $\:\dot{\mathrm{V}}{\mathrm{O}}_{2}\mathrm{p}\mathrm{e}\mathrm{a}\mathrm{k}$, the reported computations open doors for further investigation on sensitivity, where the weight of each of the input parameters on DLO_2_, DMO_2_ would be estimated. As expected, PAO_2_ was the primary factor affecting DLO_2_ at any altitude, yet interestingly its weight remained around 45% up to 5500 m and then increased largely at higher altitude (Fig. 6 panel A). This observation is compatible with the hypothesized lung diffusion capacity reserve^33^. Interestingly, the increase in the weight of PAO_2_ occurs at the altitude where DLO_2_ reached its maximum value, corresponding when the lung diffusion capacity reserve may be depleted (and which is also the highest altitude of permanent human life on Earth). Contrarily, the weight of PaO_2_ increased in a linear manner from sea-level to ~ 7500 m and then increased more rapidly up to the altitude of Mount Everest. Interestingly, the weight of PaO_2_ also increased linearly for DMO_2_, but its relative importance compared with other parameters was lesser (below 20% at any altitude, Fig. 6B).

### Limitations

Unfortunately, most of the expeditions in Himalaya or in the Andes, included male participants only. Since females experience greater respiratory limitations during exercise than males^46, 47, 48^, the findings of the present article may apply to males only and further studies are required to estimate DLO_2_ and DMO_2_ in females.

The present article is a theoretical analysis of the O_2_ transport cascade at altitude, which focused on the two diffusion steps. These steps are very difficult to measure either in vivo or in vitro and even more during field studies such as expeditions to high altitude. Yet hopefully the future advances in measuring techniques will allow researchers to report valuable measures of DLO_2_ and DMO_2_ at altitude. The carbon monoxide diffusion capacity (DLCO) method has been proposed to estimate DLO_2_ but is not yet fully operational at altitude and the NIRS method has been proposed to estimate DMO_2_^28^, but field studies using this technique remain to be performed.

The macroscopic model used in this study is based on Fick’s equation and Fick’s principle of mass conservation which are widely used in altitude literature^3, 49^, but it does not consider refined parameters for local oxygen diffusion. For example, during maximal exercise, increases in body temperature and blood acidosis (due to changes in venous CO_2_ pressure and lactate concentration) modify the blood-oxygen dissociation curve and may have a significant impact on both DLO_2_ and DMO_2_. More elaborated models for the O_2_ dissociation curve including effects of temperature, PCO_2_, pH, 2-3DPG and the CO_2_ dissociation curve^50, 51, 52^ would make computations of DLO_2_ and DMO_2_ more accurate^17^. The O_2_ / CO_2_ / H^+^ interactions with haemoglobin and O_2_ diffusion cannot be accounted for by the variations of p50 as they are presented herein. Additionally, the macroscopic material balance presently used assumes that both the capillary and the lung/muscle act as a perfectly mixed compartments, meaning there is no spatial non-homogeneity in concentration. Considering the potential non-homogeneity in concentration may impact DLO_2_ and DMO_2_^17^. Spatial heterogeneity (i.e. significant portion of the tissue will be well above zero PO_2_ even if some areas are hypoxic) within muscle will narrow under the decrease in PO_2_ distribution with altitude, resulting in a lower value of PmitO_2_, as previously reported at maximal exercise^53^. However, the lack of reliable data from field studies is critical and unfortunately limits the use of such comprehensive models. Further work is needed to fully assess the impact of the above-mentioned parameters on DLO_2_ and DMO_2_ using more comprehensive modelling of local O_2_ diffusion.

By definition, changes in PmitO_2_ alter the prediction of DMO_2_. The present study used the classical assumption in altitude physiology of a negligible PmitO_2_ but also repeated computations for non-negligible PmitO_2_, which resulted in greater DMO_2_ and with a maximum reached at a higher altitude than DMO_2_ estimated from a negligible PmitO_2_. However, the shape of the curve describing DMO_2_ as a function of altitude did not change.

The present computation of DLO_2_ did not distinguish the alveolar-capillary diffusion limitation and ventilation-perfusion inequality and/or shunt. Therefore, the impact of high-altitude pulmonary oedema or ventilation-perfusion inequality cannot be assessed and may be significant, particularly at the highest altitude levels. Literature on this topic is extensive and further studies using for example the multiple inert gas elimination technique^54^ are needed to differentiate the effects of alveolar-capillary diffusion limitation from the effects of high-altitude pulmonary oedema or ventilation-perfusion inequality. Previous publications reported field measurements in the arterial blood of humans at extreme altitudes. The calculated alveolar–arterial oxygen difference indicated a degree of functional limitation in pulmonary diffusion or subclinical pulmonary edema and/or ventilation-perfusion mismatch, which may explain the values for PaO_2_ lower than expected^55^.

Additionally, other confounding factors may have introduced noise in the current dataset based on various publications from various groups over the past century of research, namely: the use of supplemental oxygen during ascent^55^, dehydration or drugs such as acetazolamide, inhaled salmeterol, dexamethasone, sildenafil, or tadalafil may have altered the measured/estimated values reported^56^.

### Clinical implication

The estimation of both DLO_2_ and DMO_2_ values using simple laboratory techniques (e.g., metabolic cart, thoracic bio-impedance, and an arterialized blood sample) both at altitude and in several diseases may shed light on the role of the two diffusion steps of the O_2_ transport cascade and help medical advice as to whether treatments should focus on diffusion or not.

## Conclusion

The present study combined the estimation of both the lung and the muscle diffusion capacities, and their estimations were based both on literature data. Simulated ascent to Mount Everest revealed that, on the one hand, DLO_2_ increased from sea-level to 5500 m and then decreased yet remaining higher than sea-level values. Such variations were compatible with the lung diffusion capacity reserve. On the other hand, DMO_2_ increased from sea-level to 3500 m and then decreased to a larger extent, reaching at high altitude values lower than the sea-level value (Both altitudes determined with PmitO_2_ set at 0 mmHg and p50 set at 26.6 mmHg). These variations were also compatible with the muscle diffusion capacity reserve, yet the muscle reserve seems depleted at a lower altitude than the lung reserve.

## Data Availability

Raw results of the computations presented in this article are available upon reasonable request at nicolas.bourdillon@unil.ch.
